# Circulating tumor DNA profiling for non-invasive genomic analysis in Indian lung cancer patients: A real-world experience

**DOI:** 10.1016/j.jlb.2025.100300

**Published:** 2025-05-21

**Authors:** Prerana Jha, Rohit Mishra, Asim Joshi, Neha Sharma, Minit Shah, Govind Babu, Amit Rauthan, Sewanti Limaye, Nandini Menon, Venkataramanan Ramachandran, Vanita Noronha, Prashant Kumar, Kumar Prabhash

**Affiliations:** a Karkinos Healthcare Pvt Ltd, Navi Mumbai, Maharashtra, 400705, India; b Karkinos Foundation, Mumbai, Maharashtra, 400086, India; c Homi Bhabha National Institute, Training School Complex, Anushakti Nagar, Mumbai, 400094, Maharashtra, India; d Department of Medical Oncology, Tata Memorial Hospital, Mumbai, 400012, Maharashtra, India; e HCG Cancer Hospital, Bengaluru, 560027, India; f Manipal Hospital, Bengaluru, Karnataka, India; g Department of Medical and Precision Oncology, Sir HN Reliance Foundation Hospital and Research Centre, Mumbai, 400004, Maharashtra, India; h Centre of Excellence for Cancer – Gangwal School of Medical Sciences and Technology, Indian Institute of Technology Kanpur, Kanpur, Uttar Pradesh, 208016, India

**Keywords:** Lung cancer, Liquid biopsy, ctDNA sequencing, Diagnosis, EGFR

## Abstract

**Background:**

Liquid biopsy assays are an important tool for non-invasive detection of genetic alterations, providing an effective alternative to traditional tissue biopsies. The study aimed to investigate the utility of ctDNA based next generation sequencing for clinical management of lung cancer patients from India.

**Methods:**

We conducted ctDNA targeted sequencing on 425 lung cancer patients from India using 50 gene oncomine precision assay. The assay was validated employing 7 controls and 77 clinical samples, and the performance of the assay was evaluated. The concordance analysis with matched tissue biopsy samples was performed on 162 cases.

**Results:**

Among the 425 lung cancer samples, 47 % harbored at least one mutation. *EGFR* was the most frequently altered gene (25.2 %), followed by *TP53* (19.8 %) and *KRAS* (4.5 %). Concordance with tissue biopsy data was 77 % for *EGFR*, 79 % for *TP53* and above 97 % for low frequency mutations. The assay demonstrated 100 % specificity and around 60 % sensitivity for the majority of clinically relevant genetic alterations including *EGFR, KRAS* and *BRAF*. Notably ERBB2 alterations were detected with 100 % sensitivity and specificity.

**Conclusion:**

The ctDNA assay demonstrates high accuracy and specificity, for both prevalent and rare genetic alterations. While further advancements are needed to enhance sensitivity and routine clinical application, our ctDNA profiling assay offers a reliable alternative for detecting genetic alterations in lung cancer patients, with significant potential for clinical integration in the Indian healthcare context.

## 1 Introduction

The advent of precision medicine has revolutionized lung cancer diagnosis and treatment [1]. Personalized therapeutic strategies based on the genomic profiles of individual patients have become the standard of care for lung cancer patients. In recent years, liquid biopsy has emerged as a pivotal tool alongside tissue biopsies in the clinical management of lung cancer [[2], [3], [4]]. In particular, the characterization of circulating tumor DNA (ctDNA) provides a dynamic and comprehensive view of the lung cancer genome, enabling real-time monitoring of tumor evolution and response to therapy [5,6]. As a result, the International Association for the Study of Lung Cancer recommends the use of liquid biopsies in cases where tissue specimens are inaccessible, inadequate, and repeat biopsies are warranted [7,8]. Importantly, liquid biopsy holds immense potential for the genomic profiling of tumors and detection of genetic alterations in key oncogenes, such as *EGFR*, *KRAS*, and *BRAF* enabling timely identification of therapy resistance, residual disease, and recurrence [9]. However, comprehensive genomic profiling of lung cancer patients remains limited to mutational profiles obtained from tissue biopsies [[10], [11], [12], [13]]. Moreover, despite its promise, the integration of liquid biopsy into routine clinical practice remains limited due to challenges such as lack of standardized protocols, limited clinical validation across diverse populations, and concerns about assay sensitivity—particularly given the low quantity and fragmented nature of DNA/RNA typically extracted from these samples. Next-generation sequencing (NGS) offers key advantages in this context, enabling highly sensitive, multiplexed detection of genetic alterations from minimal input material. NGS on liquid biopsies facilitates minimally invasive, real-time genomic profiling of tumors, enabling early identification of resistance-associated alterations and informing more tailored, evidence-based therapeutic strategies. In this study, we present a ctDNA-based profiling using NGS on lung cancer samples. The assay enables detailed characterization of genetic alterations in a non-invasive manner, providing a robust tool for the molecular characterization and identification of therapeutic targets in lung cancer. We present the clinical application of liquid biopsy assay comprising the genomic profiles of 425 lung cancer patients. Notably, this is one of the first targeted NGS study published on a large cohort from India and sheds light on paramount importance of liquid biopsy in clinical management of lung cancer patients. The primary aim of this study was twofold [1]: to develop and validate a ctDNA sequencing assay tailored for Indian cancer patients and [2] to generate real-world data of targetable mutations prevalent in Indian lung cancer patients, providing crucial insights into the prevalence of mutations like EGFR and demonstrating the assay's utility in clinical practice.

## 2 Materials and methods

### 2.1 Blood sample collection and patient cohort

We analyzed targeted sequencing data from blood of 425 non-small cell lung cancer (NSCLC) patient samples received for diagnostic purposes between March 2023 and September 2023. The study protocol was reviewed and approved by the ethical committee of Karkinos Healthcare under Protocol No. KH-IEC/02/2024. It was a multicentric study where blood samples were collected in PAXgene Blood ccfDNA Tubes (Qiagen). Our inclusion criteria to process the samples were 1) availability of minimum 10 ml of blood sample 2) non-haemolysed samples and 3) ctDNA concentration >5 ng to ensure adequate library preparation and downstream analysis. The exclusion criteria included samples collected within15 days of therapy initiation.

For sample administration, around 10 ml of blood was collected into the PAXgene Blood ccfDNA Tube (Qiagen). The sample was immediately shipped at 4 °C to preserve its integrity. Plasma was subsequently isolated and stored at −80 °C until cfDNA extraction. The cfDNA was stored at −80 °C until further processing. The integrity of the cfDNA was assessed using a TapeStation system to check for integrity and potential genomic DNA contamination. Only samples with >90 % cfDNA purity were used for subsequent analyses.

The ctDNA was isolated from all the blood samples and subjected to targeted sequencing using a 50-gene panel at our institution. Detailed clinical and demographic information, including gender, age, disease stage, treatment history, smoking status, and PD-L1 expression, was obtained for each patient.

### 2.2 ctDNA extraction, sequencing and analysis

For ctDNA extraction, approximately 10 ml of peripheral blood was processed to separate plasma. Cell Free DNA/TNA was extracted from around 4–6 ml of plasma using the Qiagen Circulating Nucleic Acid isolation kit. Libraries were prepared from input ctDNA varying from minimum 5 ng to 26.6 ng ng of cfDNA/cfTNA for control and clinical samples. AmpliSeq-based targeted sequencing utilizing a panel of 50 genes (Oncomine Precision Assay) was conducted on all samples. This NGS gene panel encompassed 3159 distinct variants, including single nucleotide variants (SNVs), indels (insertions and deletions), fusions, and copy-number variations (CNVs).

The panel underwent in-house validation in accordance with College of American Pathologists (CAP) and National accreditation board for testing and calibration laboratories (NABL) guidelines, using controls and orthogonally validated clinical samples as.

(detailed in Supplementary materials and methods). Sequenicng was performed on Genexus Sequencer (Thermo Scientfic). Analysis was performed using Ion Torrent Genexus Software 6.6.2.1, aligning raw data to the human genome Hg19 and generating variant calling files (VCFs). Subsequently, VCF files underwent downstream variant prioritization based on several parameters, including variant allele frequency ≥5 %, Phred quality score >50 for SNVs, ≥50 read counts for fusion genes, and for CNVs, amplification with 6 copy numbers and CNV ratio >2, as well as deletions or copy-number loss with CNV ratio <0.85 and MAPD score <0.5 to prioritize high-quality variants. Additionally, variant heatmap and correlation analysis with various clinical parameters were performed using the maftools package in R.

### 2.3 Statistical analysis

For comparative analysis of gene alterations across different patient groups with clinicopathological features, Maftools (version 48) was utilized. Various features, including tumor histology, patient gender, age, metastasis status, treatment history, and smoking history, were examined. A minimum sample count of 3 in each group was set as the cutoff for comparison. Statistical significance was determined using Fischer's exact test, with a p-value <0.05 considered significant. The results of the comparative analysis were visualized using co-barplots generated using Maftools in R.

## 3 Results

### 3.1 Clinical characteristics of the lung cancer patients

**Fig. 1:**
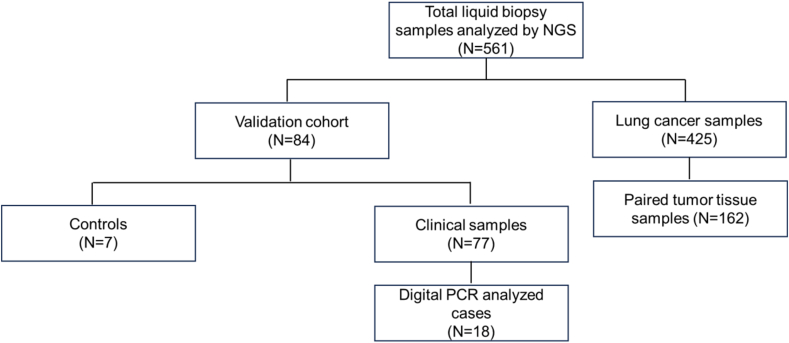
**Overall structure of the study.** Of the total 561 samples, 425 were lung cancer samples which were to describe the mutational profiles of Indian lung cancer patients. Performance of the assay was computed based on concordance analysis with matched 162 tumor tissue biopsy data.

We employed liquid biopsy based NGS testing on a total of 561 cancer samples (Fig. 1). Amongst these, liquid biopsy samples from 425 lung cancer patients were collected for targeted sequencing between March and September 2023 as detailed in Table 1. Males accounted for 63 % (n = 270) of patients and females constituted 27 % (n = 155). The average age of diagnosis of lung cancer patients was 56 years. There was an equal distribution of smokers and non-smokers within our cohort. Complete clinical and histological characteristics of the patients are detailed in Supplementary Table S1.

**Table 1:** Demographic and clinicopathological details of the cohort.

Characteristic	Category	Number of patients
Gender	Male	270
	Female	155

Age Group	<45	74
	>45	351

Smoking status	Non-smoker	117
	Smoker	118
	NA	190

Histological type	Adenocarcinoma	291
	Lung squamous carcinoma	52
	Adenosquamous	4
	Pleomorphic	1
	NA	77

### 3.2 Liquid biopsy based genomic profiling of Indian lung cancer patients

**Fig. 2:**
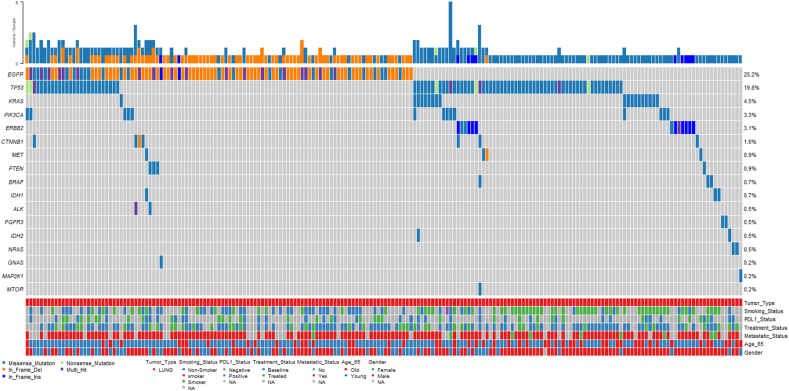
Heatmap representation of genetic alterations observed in 425 lung cancer patients. A total of 47 % patients harbored mutations including SNVs and INDELS. The clinicopathological characteristics of patients are color coded and indicated at the bottom of the heatmap.

**Fig. 3:**
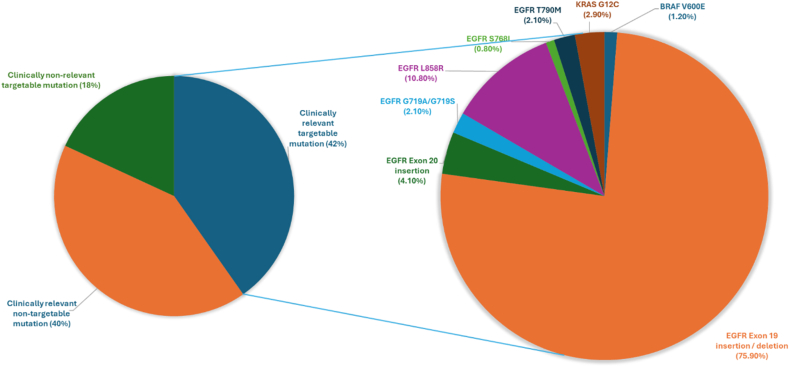
Distribution of the variants obtained based on ctDNA profiing of 425 patients. The variants obtained were categorized into targetable, non-targetable and clinically non-relevant and are depicted in the form of pie-chart. The distribution of targetable alterations is shown on the pie chart on the right side.

The analysis of targeted sequencing data from 425 NSCLC samples revealed a total of 1389 genetic alterations, encompassing point mutations/indels, copy number variations, and fusions, distributed across 20 genes (Fig. 2, Supplementary Table S2). Overall, 47 % of patients harbored at least one mutation among the 50 genes tested. *EGFR* emerged as the most frequently altered gene, consistent with patterns observed in Asian lung cancer patients, with a prevalence of 25.2 %. The EGFR alterations included the Exon 19 deletion and the L858R point mutation in Exon 21, constituting 14.4 % and 5.4 % across 425 cases respectively. Notably, EGFR-TKI resistance-associated mutations, including T790M, were observed in 5 patients. Additionally, a number of other rare EGFR variants, beyond the common activating mutations, were observed and are detailed in Supplementary Fig. S1. In addition, EGFR mutations were significantly common in females than males (p < 0.001) (Supplementary Fig. S2). *TP53* ranked as the second most frequently altered gene, with a frequency of 19.8 %. Among other commonly detected mutations in lung cancer patients, *KRAS* mutations were identified in approximately 4.5 % of cases, while EML4-ALK fusions were detected in only 0.5 % of patients, suggesting potential limitations of ctDNA profiling in detecting fusions. Furthermore, genetic alterations in *ERBB2* were observed in 3.1 % of cases, *PIK3CA* mutations at a frequency of 3.3 %, and *CTNNB1* alterations in 1.6 % of cases and alterations in *BRAF* were identified at a frequency of 0.7 %. Our analysis also revealed low frequencies of alterations in several other genes: *MET* (0.9 %), *PTEN* (0.9 %), *IDH1* (0.7 %), *FGFR3* (0.5 %), *IDH2* (0.5 %), *NRAS* (0.5 %), *GNAS* (0.2 %), *MAP2K1* (0.2 %), and *MTOR* (0.2 %). Overall, a total of 177 patients harbored targetable mutations, 171 patients had non-targetable mutations and 77 patients did not possess clinically relevant mutations as shown in Fig. 3.

### 3.3 Concordance of liquid vs tissue biopsy in lung cancer

**Fig. 4:**
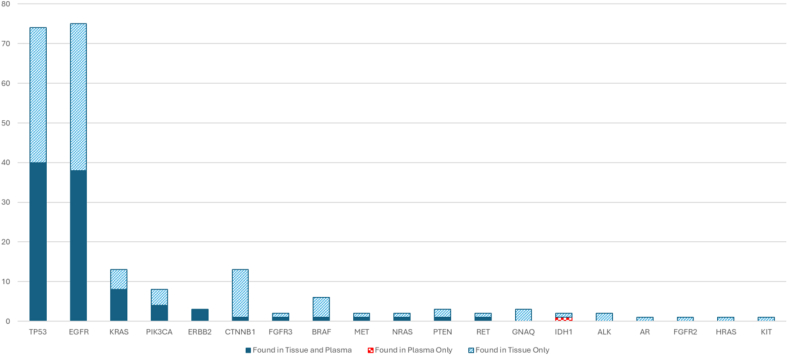
Concordance analysis of the data. The bar graph represents the concordance between solid and liquid biopsy data based on analysis of 162 samples with availability of both solid and liquid biopsy profiling data.

We analyzed the performance of our assay by evaluating the concordance with data from matched tissue samples (Fig. 4, Supplementary Table S3). Tissue and liquid biopsy data was available from 162 patients. Genes like *ERBB2, FGFR3, MET, NRAS, RET, AR, HRAS, IDH1, KIT, FGFR2, PIK3CA, PTEN, BRAF, KRAS and CTNNB1* exhibited concordance rates above 97 %. A higher concordance rate for these may be contributed to the smaller cohort for these frequencies. The concordance among genes harboring higher frequency mutations including *EGFR* and *TP53* have concordance rates at 77.16 % and 79.01 %, respectively. However, for commonly known activating *EGFR* mutations, the concordance was observed to be 88 %. We then calculated the sensitivity and specificity of our assay. Among the genes analyzed, *ERBB2* displayed 100 % sensitivity, specificity, PPV, and NPV, indicating flawless detection in this dataset. Conversely, *EGFR* and *TP53*, showed 100 % specificity, had sensitivity of 50.67 % and 54.05 % respectively, suggesting a notable proportion of false negatives. This is mainly attributed due to lower cfDNA concentration in samples. The commonly observed EGFR mutations including exon 19 deletions, L858R and G719 A/S were detected with a sensitivity of 60 %. *KRAS* also demonstrated high specificity (100 %) and a sensitivity of 61.54 %, with similar trends observed for *PIK3CA*, *FGFR3*, *MET*, *NRAS*, and *RET*, each showed 100 % specificity, however, sensitivities varied around 50 %.

## 4 Discussion

The successful treatment of lung cancer patients relies on the accurate identification of genetic alterations. Although tissue biopsy-based genetic mutation detection is the gold standard, it is often invasive, risky, and sometimes not feasible due to tumor location, metastatic disease, or insufficient tissue samples [14]. Moreover, advanced healthcare facilities required for performing biopsies are scarce, especially in resource- and expertise-limited countries like India [15,16]. Therefore, a ctDNA profiling assay tailored for the Indian healthcare context addresses critical needs by simplifying sample collection, even in less specialized settings, and offering a cost-effective alternative to traditional tissue biopsies. In this study, we aim to validate the clinical utility of a ctDNA profiling assay for the management of lung cancer patients. Through robust analytical validation, our assay demonstrated high accuracy and specificity across various mutation classes, including SNVs, and INDELs. In a cohort of 425 NSCLC patients, the ctDNA assay identified a total of 1389 genetic alterations, with 47 % of patients harboring at least one mutation among the 50 genes tested. *EGFR* mutations were the most prevalent, found in 25.2 % of cases, with Exon 19 deletions and L858R mutations being the most common. This aligns with the mutation patterns typically observed in Asian lung cancer patients [17,18]. Other notable mutations included *TP53* (19 %), *KRAS* (4.5 %), ERBB2 (3.1 %), *PIK3CA* (3.3 %), and EML4-ALK fusions (0.5 %), with lower frequencies observed for *MET, PTEN, IDH1, FGFR3*, and other genes.

Data from tissue biopsy and paired blood samples from 162 patients demonstrated higher mutation concordance rate above 97 % for *ERBB2, FGFR3, MET, NRAS, RET, AR, HRAS, IDH1, KIT, FGFR2, PIK3CA, PTEN, BRAF, KRAS* and *CTNNB1*. The frequency of these mutations was low in the present study and their concordance rate may not be reflective of cfDNA selective detection rate of particular mutation type. In EGFR and TP53, with higher frequency in the lung cancer cohort, we observed the concordance rates of 77.16 % and 79.01 %, respectively. The lower detection rate of theses variants in plasma could be mainly due to lower release of cfDNA in some case, thereby reducing the detectability in blood. The concordance for commonly known activating EGFR mutations was 88 %, indicating better performance for specific clinically relevant mutations, and strongly suggests that cfDNA can assist in treatment decisions of majority cases, where at times the tumor tissue cannot be obtained.

The ctDNA assay exhibited 100 % sensitivity, specificity, PPV, and NPV for ERBB2, affirming its reliability in detecting this targetable mutation. Conversely, sensitivity observed for *EGFR* and *TP53* was 50.67 % and 54.05 %, indicating a notable proportion of false negatives, likely due to low ctDNA abundance or technical detection limitations. The performance of our assay for EGFR mutations is comparable to other assays evaluated across multiple studies [[19], [20], [21], [22]]. The study by Douillard et al., showed the EGFR mutation status concordance between 652 matched tumor and base line plasma samples as 94.3 % and test sensitivity of 65.7 % and test specificity as 99.8 % [22]. The noninterventional diagnostic ASSESS study (NCT01785888) evaluated 1162 matched samples for utility of ctDNA from plasma for EGFR mutation testing and showed the concordance of mutation as 89 % (sensitivity 46 % and specificity 97 %) [20]. In study by Lin et al., Tissue-NGS identified 74 clinically relevant mutations, including 52 therapeutic targets, at sensitivity of 94.8 %, while plasma-NGS detected 41 clinically relevant mutations and showed the sensitivity of 52.6 % (*p* < 0.001) [23]. The concordance between plasma and tissue testing was found to be 82.9 % (95 % confidence interval [CI]: 77.55, 87.45). The sensitivity and specificity of NGS were 68.4 % (95 % CI: 56.92, 78.37) and 90.1 % [95 % CI: 84.36, 94.21), respectively. The Indian study conducted on 245 metastatic NSCLC patients for concordance of EGFR mutation by ctDNA versus tissue biopsy showed concordance of 82.9 % between plasma and tissue testing, sensitivity and specificity of NGS were 68.4 % and 90.1 %, respectively [24]. Ours is the largest cohort of ctDNA analysis of lung cancers from Indian subset. The present study showed comparable concordant sensitivity and specificity, however a slightly lower concordance for EGFR mutation, as compared to Western literature may be attributed to factors, such as prior therapeutic treatment given for EGFR mutant tumors and the technical limitations with lower ctDNA components in blood in some case. For other prevalent actionable mutation in lung cancer *KRAS*, *PIK3CA*, *FGFR3*, *MET, NRAS*, and *RET* demonstrated 100 % specificity with varied sensitivities around 50 %. EML4-ALK fusions, RET rearrangements, ROS1 fusions, and NTRK1/2/3 fusions are observed at a cumulative frequency of 3 %–10 % in lung cancers. However, we identified ALK fusions in only 2 cases, which could be mostly attributed to the shorter time period for stability of RNA fragments in blood and presence of molecules less than the detection capability of the assay. A better method for detecting fusion variants in ctDNA could be the DNA based fusion detection. Tissue based NGS has shown significantly higher sensitivity and accuracy across studies, both in newly diagnosed and treated patients. Our findings strongly indicate that ctDNA positive diagnosis can be confidently taken for clinical treatment management, however, the cases where ctDNA negatives are suggestive of tissue based molecular diagnostics.

Overall, the ctDNA profiling assay demonstrates substantial promise for clinical management of lung cancer patients. The assay's high specificity and PPV underscore its potential as a reliable non-invasive diagnostic tool, although further advancements in the technology is warranted to improve sensitivity for routine implementation in clinical practice. This study supports the integration of ctDNA assays into standard diagnostic workflow to complement traditional tissue biopsies, providing an alternative approach for longitudinal monitoring and disease management. However, there remain certain barriers to the implementation of liquid biopsy-based assays in clinical practice. Primarily, challenges related to sample collection and processing, such as ensuring that genomic DNA contamination is avoided and that blood samples are not hemolyzed, can affect the accuracy of the assay. Additionally, the effects of recent chemotherapy may cause the mutational profile in the liquid biopsy to differ from that of the tumor. The release of tumor DNA into the bloodstream is also variable, which can affect the sensitivity and specificity of the assay, occasionally leading to false negatives. Furthermore, for NGS assays performed at very high depths, there is a risk of PCR-induced errors and false positives. To mitigate these errors, a Unique Molecular Index (UMI)-based PCR method was employed in our NGS panel. In conclusion, while liquid biopsy offers a valuable method for mutation detection, particularly in cases where tissue biopsies are small or difficult to obtain, proper methodology, accurate sample collection, and thorough assay validation are crucial.

## Availability of data and materials

The sequencing data of the patient is not available publicly due to patient confidentiality.

## Authors' contributions

PK and KP conceptualized and designed the entire study. PJ, RM and AJ analyzed the data. RM, AJ and NS parsed the clinicopathological reports. PJ and NS performed experiments. PJ, RM and AJ wrote the manuscript. PJ, NS, MS, GB, AR, SL, NM, VR, VN, KP and PK critically reviewed and edited the manuscript. All authors have read and approved the final manuscript.

## Ethics approval, patient's consent to participate and publication

The study protocol was reviewed and approved by the ethical committee of Karkinos Healthcare (Protocol No KH-IEC/02/2024).

## Data availability

All the supporting data are available upon request.

## Funding

This study did not receive any external funding.
