# Supplementary material for: Circulating tumor DNA profiling for non-invasive genomic analysis in Indian lung cancer patients: A real-world experience
Source: J Liq Biopsy. 2025 May 21;8:100300. doi: 10.1016/j.jlb.2025.100300 (PMC12158504; doi:10.1016/j.jlb.2025.100300)
Supplement: Multimedia component 2 [file mmc2.docx]

**Supplementary File**

**Materials and Methods**

**Assay Validation**

The validation was performed for Oncomine Precision Assay used for liquid biopsy, as per the CAP and Association of Molecular Pathology (AMP) joint consensus guidelines for somatic mutation detection using next generation sequencing (NGS). A total of 7 controls (Supplementary Table S4) and 77 clinical blood samples, including 18 tumor-matched samples, were used. The clinical details and concentration of cfDNA and amount used for the assay is specified in (Supplementary Table S5). The performance characteristics of the assay were evaluated for analytical accuracy, variant calling accuracy, analytical sensitivity, precision (within run repeatability) and reproducibility (across run precision). The following formulas were used in this analytical validation,

PPV = TP/(TP+FP);

NPV = TN/(TN+FN);

Sensitivity (PPA) = TP/(TP+FN);

Accuracy = (TP+TN)/(TP+TN+FP+FN);

Specificity = TN/(TN+FP);

Repeatability/Reproducibility = (TP)/(TP+FN+FP).

[True Negative (TN) False Negative (FN) False Positive (FP) True Positive (TP)].

Intra-assay (within run) precision was assessed using clinical samples analyzed in triplicate in one run by one operator. Inter-assay (across run) reproducibility was assessed using clinical samples analysed with one replicate each across 3 different runs by at least two different operators over multiple days.

**Digital PCR (dPCR)**

We performed dPCR for EGFR gene using QuantStudioTM Absolute QTM digital PCR (Applied Biosystems) system. Two EGFR multiplex panels (Driver Mutation panel and Drug Resistant Mutations panel) targeting various mutant alleles were used. Driver mutation panel covered p.E746_A750del and L858R/L. The drug resistant mutations panel covered T790M/L, C797S, L792F and L718Q. Each multiplex panel has a single VIC wild-type probe and the mutant probes had combination of FAM, ABY and JUN fluorophores. Candidate assays were screened in duplex dPCR with total 16 samples including one wild type EGFR variant on NGS and variant allele frequency ranging from 0.1% to 12%. The input ctDNA used for dPCR ranged from as low as 5ng- 30ng. The PCR was performed as per manufacturer’s protocol.

**Results**

**Clinical Assay validation**

A total of 23 chips and 87 unique libraries were sequenced as part of the analytical validation. Analytical accuracy was assessed with 2 HapMap samples: NA12878 and NA19240. A combination of controls and clinical samples were assessed for variant calls and true positive (TP), false positive (FP) and false negative (FN) variant calls were further confirmed via an orthogonal digital PCR methodology (Supplementary Tables S4 and S5). Sensitivity (PPA) and Positive Predictive Value (PPV) were characterized for each variant class. For 16 selected cases with available ctDNA, digital PCR (Thermo) was performed for EGFR targets and concordance of results are shown in Supplementary Table S6.

The analytical sensitivity was determined for SNV/INDELs and CNVs using Acrometrix Control Plasma A-C, which harbors 9 SNV/INDELs and 2 CNVs detected by the assay. As shown in Table 1, the limits of detection for SNV, MNV and INDELs was 0.5% allele frequency (AF) and CNVs >4 copies. We observed overall analytical accuracy of 99.57% and the analytical specificity of 99.95% and 100%, for SNV/INDELs and CNVs respectively. The analytical sensitivity for SNV/INDELs decreased with reduction in allele fraction and was observed to be 100% at 1% AF, 78% at 0.5% AF, and 44% at 0.25% AF. The analytical sensitivity of the panel was also determined for SNV/INDEL and CNV using Seracare ctDNA complete mutation mix which harbors 16 SNV/INDELs and 2 CNVs detected by the assay. For SNV/INDELs, the analytical sensitivity was 100% at 5% AF, 2.5% AF and 0.5% AF and 63% at 0.1% AF. Variants not detected at 0.5% were attributable to minimum molecular coverage requirement of 3 for variant calling. For CNVs we observed 100% sensitivity at 4 copies.

Limit of input study was performed using standard input of 20 ng and higher input at 50 ng with Seracare ctDNA. We observed similar results using both 20 and 50 ng input. Variants not detected were attributable to minimum molecular coverage requirement of 3 for variant calling. For CNV variants we observed 100% sensitivity at 4 copies (CNV Ratio of 2) for both 20 and 50 ng input. Input amount for clinical plasma samples tested during validation ranged from 4.52 ng/uL to 26.6 ng/uL with 20 uL total volume. Although the recommended input amount is 26.6 ng, we were able to successfully validate samples with an input amount of as low as 4.5 ng. The lowest AF that was orthogonally confirmed at 5 ng input was 0.5% for INDEL.

Overall variant calling accuracy was evaluated using plasma samples. Variants in the assay was classified as TP, FP, FN, or TN with truth defined orthogonally comparing against the solid tumor data generated from same clinical samples for a total of 18 clinical samples. For SNV/INDELs, overall variant calling accuracy was 100%. The PPV was 100% for VAF >0.5%. For CNVs, overall variant calling accuracy and PPV were 100%. We obtained 100% concordance for SNV/INDELs on evaluation of the intra-assay repeatability and inter-assay reproducibility.

Table 1: Performance of the ctDNA profiling assay

| **Component** | **SNV/INDEL** | **CNV** |
| --- | --- | --- |
| **Threshold** | ≥0.5% AF | ≥4 Copies |
| **Analytical Accuracy** | 99.3% | |
| **Analytical Specificity** | 99.98% | 100% |
| **Analytical Sensitivity** | 78% | 100% |
| **Variant Calling Accuracy** | 100% PPA and PPV | 100% PPA and PPV |
| **Repeatability** | 100% | 100% |
| **Reproducibility** | 100% | 100% |
