# Supplementary material for: Circulating tumor DNA profiling for non-invasive genomic analysis in Indian lung cancer patients: A real-world experience
Source: J Liq Biopsy. 2025 May 21;8:100300. doi: 10.1016/j.jlb.2025.100300 (PMC12158504; doi:10.1016/j.jlb.2025.100300)
Supplement: Multimedia component 3 [file mmc3.docx]

Supplementary Figure S1


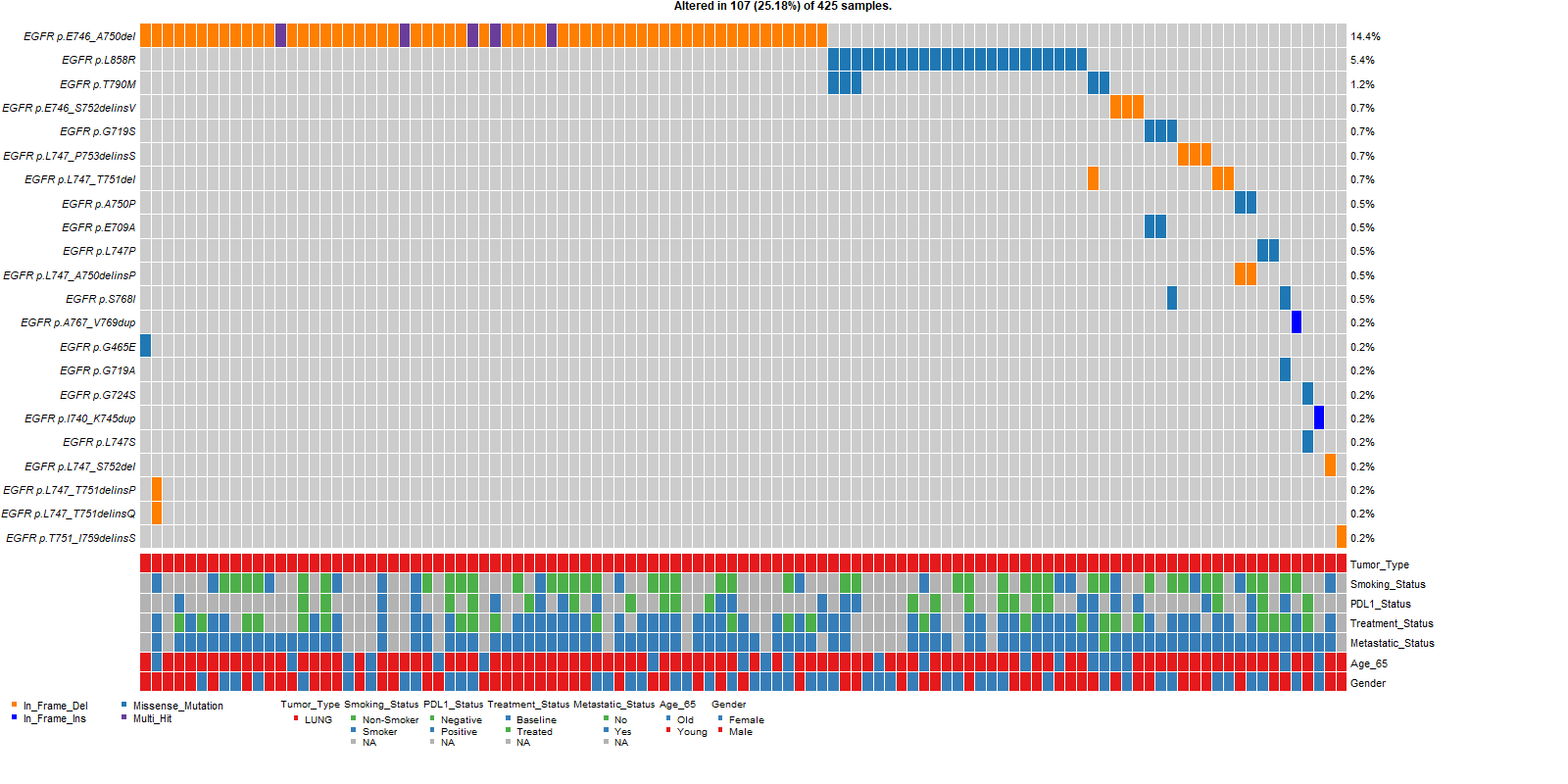


Supplementary Figure S1: Heatmap representation of EGFR mutations. All the EGFR mutations obtained after profiling of 425 lung cancer patient are depicted in the heatmap. Only EGFR mutations are shown in the heatmap. Patients wildtype for EGFR mutations are not shown. The clinicopathological characteristics of patients are color coded and indicated at the bottom of the heatmap.

Supplementary Figure S2

Supplementary Figure S2: Clinical corelation of genetic alterations. The genetic alterations obtained were corelated with the clinical parameters of the patients including gender and smoking habits and are shown in the form of bar chart.
